# Design, Synthesis and Evaluation of Novel Tacrine-Ferulic Acid Hybrids as Multifunctional Drug Candidates against Alzheimer’s Disease

**DOI:** 10.3390/molecules21101338

**Published:** 2016-10-11

**Authors:** Yingbo Fu, Yu Mu, Hui Lei, Pu Wang, Xin Li, Qiao Leng, Li Han, Xiaodan Qu, Zhanyou Wang, Xueshi Huang

**Affiliations:** 1 Laboratory of Metabolic Disease Research and Drug Development, China Medical University, Shenyang 110001, China; fuyb@mail.neu.edu.cn (Y.F.); fly1982_2008@sina.com (X.L.); leng_qiao@yahoo.com (Q.L.); 2 Institute of Microbial Pharmaceuticals, College of Life and Health Sciences, Northeastern University, Shenyang 110819, China; muyu@mail.neu.edu.cn (Y.M.); leihui-2008@163.com (H.L.); wangpu@mail.neu.edu.cn (P.W.); hanli@mail.neu.edu.cn (L.H.); x.qu@hotmail.com (X.Q.)

**Keywords:** β-amyloid aggregation, cholinesterase, ferulic acid, metal chelator, tacrine, neuroprotection

## Abstract

Five novel tacrine-ferulic acid hybrid compounds (**8a**–**e**) were synthesized and their structures were identified on the basis of a detailed spectroscopic analysis. The activities of inhibiting acetyl cholinesterase (AChE) and butyryl cholinesterase (BuChE), reducing self-induced β-amyloid (Aβ) aggregation and chelating Cu^2+^ were evaluated in vitro. Among them, **8c** and **8d** displayed the higher selectivity in inhibiting AChE over BuChE. Moreover, **8d** also showed dramatic inhibition of self-Aβ aggregation, activity of chelating Cu^2+^ and activity against Aβ-induced neurotoxicity in Neuro-2A cells.

## 1. Introduction

Alzheimer’s disease (AD) is a chronic neurodegenerative disease, which caused 60%–70% of dementia cases of people over 65 years of age in the world. The development and progression of AD will finally result in behavioral issues and loss of self-care [1,2]. Clinically, AD is characterized by β-amyloid protein (Aβ) deposition and hyperphosphorylation of tau, which lead to the formation of senile plaques (SPs) and neurofibrillary tangles (NFTs) [3]. The actions of Aβ deposition and tau hyperphosphorylation will induce a dramatic loss of synapses and neurons as well as increase the level of choline acetyltransferase (AChE) in AD patients [4]. On the other hand, the most important biometals, i.e., copper, zinc, and iron, which critically affected the aggregation behavior of Aβ, have been verified to be involved in AD [5]. In addition, metal ion chelators have shown their efficacy in ameliorating the syndrome of AD. 

**Figure 1:**
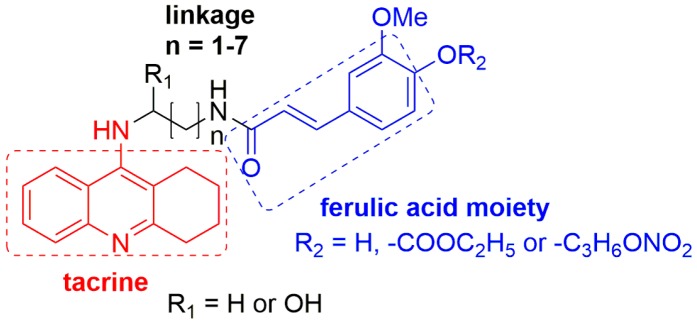
The synthesized tacrine-ferulic acid hybrids in the literature.

In view of these observations, exploring new anti-Alzheimer drugs with multiple potencies to concurrently inhibit the aggregation of Aβ and the activity of AChE will be a feasible strategy to combat AD [6,7]. To this purpose, we are prompted to exploit a series of tacrine-ferulic acid hybrids (TFAs) as anti-Alzheimer’s disease drug candidates with three potencies, including inhibiting cholinesterase (ChE), reducing self-induced β-amyloid (Aβ) aggregation and chelating Cu^2+^. These potencies are exerted by three moieties in molecules: tarcine-like heterocycle, ferulic acid moiety and piperazine fragment. In detail, tacrine is an attractive inhibitor of AChE with low molecular weight, suitable for modification [8]. As a small phenolic molecule present in fruit and vegetables, ferulic acid (FA) exhibited characteristics of inhibiting Aβ aggregation and strong antioxidant properties [9]. Several different structural families of tacrine-ferulic acid hybrids have been previously reported as potent cholinesterase inhibitors (Figure 1) [10,11,12,13], and some of them have been found to reverse scopolamine-induced cognitive impairment in vivo with low hepatotoxicity. These results suggested that tacrine-ferulic acid hybrids were potential multitarget candidates for AD therapy. Apart from tacrine and ferulic acid moieties, a piperazine fragment has the ability to bind in the mid-gorge site of AChE, which potentially contributes to inhibiting the activity of AChE [14]. Moreover, piperazine has been proposed as the chelator of bivalent metal ions, such as copper ions (Cu^2+^) [14].

In the current study, we reported the synthesis of designed TFAs and the evaluation of their activity of inhibiting cholinesterase (ChE), reducing self-induced β-amyloid (Aβ) aggregation and chelating Cu^2+^ in vitro. At the same time, neuroprotective activity of TFAs was evaluated in vitro.

## 2. Results and Discussion

### 2.1. Chemistry

**Scheme 1:**
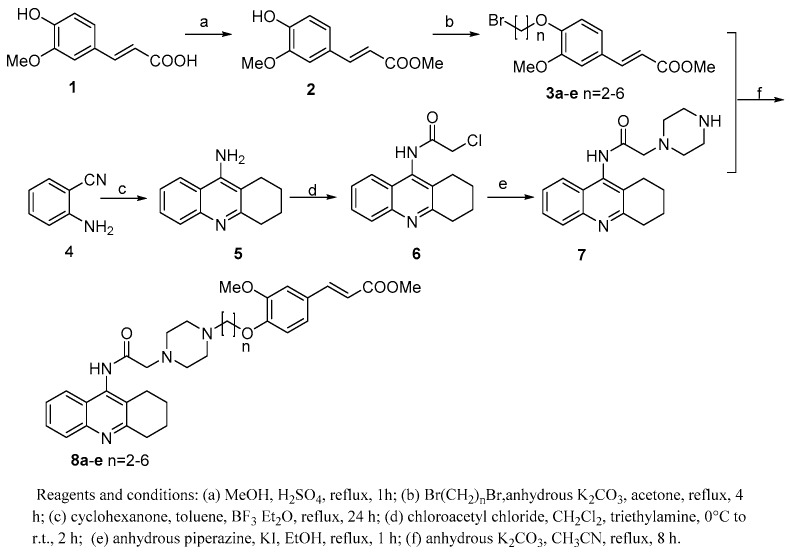
The synthetic routes to TFAs.

TFAs (**8a**–**e**) were synthesized via the routes depicted in Scheme 1. By treatment with concentrated H_2_SO_4_ and methanol, FA efficiently underwent esterification to yield a FA methyl ester **2** [15]. In order to import a straight chain alkyl (*n* = 2–6) linker at the hydroxyl of FA, **2** was then reacted with α, ω-dibromoalkanes in the presence of potassium carbonate to provide compound **3a**–**e** [16]. On the other hand, 2-aminobenzonitrile (**4**), tacrine (**5**), *N*-chloroacetyltacrine (**6**) and the key intermediate **7** were synthesized using the same methods reported by S. S. Xie, et al. [14] Finally, target compounds **8a**–**e** were obtained through nucleophilic substitution reaction between **7** and **3a**–**e**.

### 2.2. AChE and BuChE Inhibitory Activities

The inhibitory activities of TFAs **8a**–**e** against AChE and BuChE were tested based on the spectrophotometric method [17], using tacrine as the reference compound. The IC_50_ values of all tested compounds and their selectivity index for AChE over BuChE are summarized in Table 1. The results showed that all TFAs presented good inhibitory activity to both AChE and BuChE and better selectivity for AChE compared with tacrine. Especially, Compounds **8b**–**d** showed higher inhibitory activities on AChE (IC_50_ = 52.7–91.3 nM) than those of tacrine (IC_50_ = 151.3 nM). 

### 2.3. Inhibition of Self-Induced Aβ (1–42) Aggregation

TFAs **8a**–**e** were tested for their ability to inhibit self-induced Aβ (1–42) aggregation by a thioflavin T-based fluorometric assay [18], and curcumin and ferulic acid were used as reference compounds. The results (Table 1) indicated that TFAs **8a**–**e** showed potencies to a certain extent. Compared to the reference compounds, TFAs **8a**–**c** showed weaker inhibitory activities. Furthermore, TFAs **8d** and **8e** at a concentration of 20 μM exhibited similar inhibitory activities to those of curcumin and ferulic acid. On the above evidence, the length of linkage group between piperazine and FA moiety was related to the potency, and short linkage length (*n* = 2–4) decreased the flexibility of the side chain, which affected the attainment of an optimal conformation for binding to an assumed biological target.

### 2.4. Cu(II)-Chelating Properties

**Figure 2:**
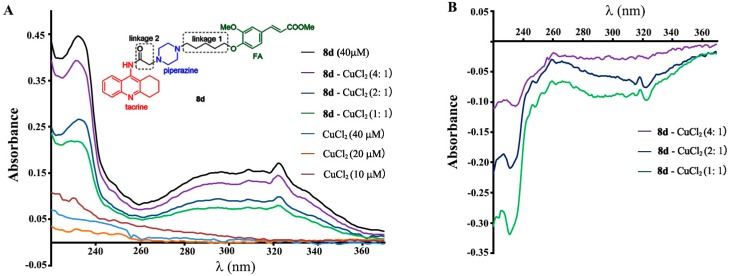
(**A**) UV (220–400 nm) absorption spectra of **8d** (40 μM) with 10, 20 or 40 μM CuCl_2_ in MeOH. (**B**) The differential spectra due to **8d**-CuCl_2_ complex formation obtained by numerical subtraction.

In recent years, there is mounting evidence to support the theory that endogenous copper can be involved as factors or cofactors in the etiopathogenesis of AD, and modulating Cu^2+^ in the brain should be to the benefit of AD treatment [19]. The chelating effect of **8d** for Cu^2+^ was studied by ultra violet (UV) spectrometry with wavelength ranging from 220 to 400 nm [20]. The results (Figure 2) indicated that there was an interaction between **8d** and Cu^2+^ and suggested that complex **8d**-Cu^2+^ had been produced.

### 2.5. Kinetic Study of AChE Inhibition

**Figure 3:**
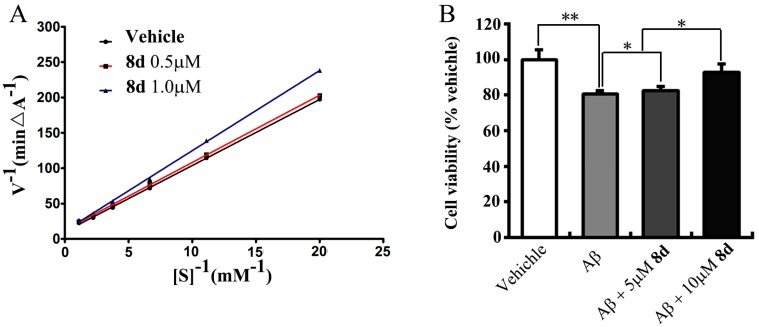
(**A**) Lineweaver–Burk plot for the inhibition of acetylcholinesterase by compound **8d**; (**B**) Effects of **8d** against Aβ-induced neurotoxicity in Neuro-2A cells. Neuroprotective effect of **8d** were evaluated by cells treated with **8d** at the concentration of 0, 5 or 10 μM in the presence of 10 μM Aβ for 48 h; Vehicle group was treated with solvent. The cell viability was assessed using the 3-[4,5-dimethylthylthiazol-2-yl]-2,5 diphenyltetrazoliumbromide (MTT) assay. Data are from three independent experiments and are expressed as the mean ± SD values. ** *p* < 0.01 compared to vehicle. * *p* < 0.05, represents significant differences from the control group.

**8d** was chosen for a kinetic study to get information on the mechanism of AChE inhibition, which is the most potent compound in this class of hybrids. Lineweaver-Burk reciprocal plots showed that both slopes and intercepts were increased when the concentration of the inhibitor increased (Figure 3A). The result indicated that TFAs were mixed-type inhibitors.

### 2.6. Protective Effects of ***8d*** on Aβ-Induced Neurotoxicity

To investigate the protective effect of **8d** against Aβ-induced cytotoxicity, **8d** (0, 5 or 10 μM) and 10 μM Aβ (1–42) was directly added into the medium of the Neuro-2A cells. The cells were incubated for 48 h. The data showed that cotreatment with **8d** led to a significant dose-dependent increase in cell viability as compared with Aβ-treated alone group (*p* < 0.05; Figure 3B).

## 3. Experimental Section

### 3.1. General Experimental Procedures

^1^H- and ^13^C-NMR spectra were recorded using a Bruker AV-600 (Bruker, Faellanden, Switzerland) and Varian Mercury 300 (Varian Inc., Palo Alto, CA, USA) spectrometers, δ in ppm rel. to tetramethylsilane (TMS), *J* in Hz. ESI MS were recorded using an Agilent 1290–6420 (Agilent Technologies, Santa Clara, CA, USA) triple quadrupole LC-MS spectrometer. Ultraviolet spectra were measured with a Beckman Coulter DU 730 nucleic acid/protein analyzer (Beckman Coulter Inc. Brea, CA, USA). Silica gel (100–200 mesh and 300–400 mesh, Qingdao Marine Chemical Ltd., Qingdao, China) were used for column chromatography. Aβ, AChE, BuChE, acetylcholine iodide, butylthicoline iodide, 5,5′-dithiobis-(2-nitrobenzoic acid) (DTNB), hexafluoroisopropanol (HFIP), and thioflavin T (ThT) were purchased from Aldrich (Sigma-Aldrich, St. Louis, MO, USA). Unless otherwise specified, all chemicals and solvents were purchased from Sinopharm Chemical Reagent Co., Ltd. (Shenyang, China).

### 3.2. Synthesis

#### 3.2.1. Procedure for the Preparation of (*E*)-Methyl 3-(4-hydroxy-3-methoxy-phenyl)acrylate (**2**)

To a solution of ferulic acid (1 g, 5.2 mmol) in 70 mL methanol, 2 mL concentrated sulfuric acid was added. The reaction solution was refluxed for 1 h and then cooled to room temperature. An amount of 200 mL ethyl acetate was added. The solution was washed by saturated aqueous solution of NaHCO_3_ three times, and saturated brine once. The organic phases were dried over Na_2_SO_4_ and concentrated under reduced pressure to give the product as yellow oil 1.01 g. The obtained oil was purified by silica gel chromatography with CH_2_Cl_2_/MeOH (10:1) as eluent to give compound **2** as yellow oil 0.64 g (Yield, 65%). ^1^H-NMR (300 MHz, CDCl_3_): δ 7.62 (1H, d, *J* = 15.6 Hz, CH = CH), 7.07 (1H, dd, *J* = 8.2, 1.8 Hz), 7.02 (1H, d, *J* = 1.8 Hz), 6.92 (1H, d, *J* = 8.2 Hz), 6.29 (1H, d, *J* = 15.6 Hz, CH = CH), 5.97 (1H, s, OH), 3.92 (3H, s, OCH_3_), 3.80 (3H, s, OCH_3_); ESI-MS *m*/*z*: 208.2 [M + H]^+^ (C_11_H_12_O_4_).

#### 3.2.2. Procedure for the Preparation of Ferulic Acid Derivatives (**3a**–**e**)

*Methyl (E)-3-(4-(2-bromoethoxy)-3-methoxyphenyl)acrylate* (**3a**): Compound **2**(500 mg, 2.4 mmol) was resolved in 30 mL acetone. An amount of 0.8 mL (9 mmol) 1,6-dibromoalkane and 600 mg (4.3 mmol) anhydrous K_2_CO_3_ was added. The solution was refluxed for 4 h and filtered. The filtrate was concentrated under reduced pressure. The obtained residue was purified by silica gel chromatography with hexane/acetone (10:1) as eluent to give compound **3a** as white solid 0.64 g (Yield, 72%). ^1^H-NMR (300 MHz, CDCl_3_): δ 7.63 (1H, d, *J* = 16.0 Hz, CH = CH), 7.09 (1H, dd, *J* = 8.0, 2.1 Hz), 7.07 (1H, d, *J* = 2.1 Hz), 6.89 (1H, d, *J* = 8.0 Hz), 6.33 (1H, d, *J* = 16.0 Hz), 4.36 (2H, t, *J* = 6.6 Hz), 3.91 (3H, s, OMe), 3.81 (3H, s, OMe), 3.68 (2H, t, *J* = 6.6 Hz); ESI-MS *m*/*z*: 315.1 [M + H]^+^ (C_13_H_15_BrO_4_).

The syntheses of compounds **3b**–**3e** were carried out by the same procedure as described for the preparation of **3a**.

*Methyl (E)-3-(4-(3-bromopropoxy)-3-methoxyphenyl)acrylate* (**3b**): white solid 0.62 g (Yield, 70%). ^1^H-NMR (300 MHz, CDCl_3_): δ 7.64 (1H, d, *J* = 16.3 Hz, CH = CH), 7.10 (1H, dd, *J* = 8.3, 1.9 Hz), 7.05 (1H, d, *J* = 1.9 Hz), 6.90 (1H, d, *J* = 8.3 Hz), 6.32 (1H, d, *J* = 16.3 Hz), 4.19 (2H, t, *J* = 6.0 Hz), 3.89 (3H, s, OMe), 3.80 (3H, s, OMe), 3.63 (2H, t, *J* = 6.0 Hz), 2.39 (2H, quint, *J* = 6.0 Hz); ESI-MS *m*/*z*: 329.5 [M + H]^+^ (C_14_H_17_BrO_4_).

*Methyl (E)-3-(4-(4-bromobutoxy)-3-methoxyphenyl)acrylate* (**3c**): white solid 0.65 g (Yield, 75%). ^1^H-NMR (300 MHz, CDCl_3_): δ 7.62 (1H, d, *J* = 16.0 Hz, CH = CH), 7.08 (1H, dd, *J* = 8.2, 2.1 Hz), 7.05 (1H, d, *J* = 2.1 Hz), 6.85 (1H, d, *J* = 8.2 Hz), 6.31 (1H, d, *J* = 16.0 Hz), 4.09 (2H, t, *J* = 6.0 Hz), 3.89 (3H, s, OMe), 3.80 (3H, s, OMe), 3.50 (2H, t, *J* = 6.0 Hz), 1.95–2.15 (4H, m); ESI-MS *m*/*z*: 343.2 [M + H]^+^ (C_15_H_19_BrO_4_).

*Methyl (E)-3-(4-((5-bromopentyl)oxy)-3-methoxyphenyl)acrylate* (**3d**): white solid 0.68 g (Yield, 79%). ^1^H-NMR (300 MHz, CDCl_3_): δ 7.64 (1H, d, *J* = 15.9 Hz, CH = CH), 7.09 (1H, dd, *J* = 8.3, 2.0 Hz), 7.05 (1H, d, *J* = 2.0 Hz), 6.85 (1H, d, *J* = 8.3 Hz), 6.31 (1H, d, *J* = 15.9 Hz), 4.06 (2H, t, *J* = 6.6 Hz), 3.90 (3H, s, OMe), 3.80 (3H, s, OMe), 3.45 (2H, t, *J* = 6.7 Hz), 1.85–2.00 (4H, m), 1.65 (2H, m); ESI-MS *m*/*z*: 357.7 [M + H]^+^ (C_16_H_21_BrO_4_).

*Methyl (E)-3-(4-((6-bromohexyl)oxy)-3-methoxyphenyl)acrylate* (**3e**): white solid 0.54 g (Yield, 58%). ^1^H-NMR (300 MHz, CDCl_3_): δ 7.64 (1H, d, *J* = 16.0 Hz, CH = CH), 7.09 (1H, dd, *J* = 8.2, 2.0 Hz), 7.05 (1H, d, *J* = 2.0 Hz), 6.85 (1H, d, *J* = 8.2 Hz), 6.31 (1H, d, *J* = 16.0 Hz), 4.05 (2H, t, *J* = 6.6 Hz), 3.90 (3H, s, OMe), 3.80 (3H, s, OMe), 3.42 (2H, t, *J* = 6.7 Hz), 1.83–1.94 (4H, m), 1.49–1.58 (4H, m); ESI-MS *m*/*z*: 371.2 [M + H]^+^ (C_17_H_23_BrO_4_).

#### 3.2.3. Procedure for the Preparation of 9-Amino-1,2,3,4-tetrahydroacridine (**5**)

To a solution of anthranilonitrile (5.0 g, 43.3 mmol) and cyclohexanone (5.0 mL) in sodium-dried toluene (110.0 mL), boron trifluoride diethyl etherate (6.4 mL) was added slowly via syringe. The mixture was then stirred at reflux for 24 h. After cooling down, the toluene was decanted, and then 120 mL 2 M NaOH was added and heated at reflux for 24 h. After cooling, the organic components were extracted with CHCl_3_, the organic layers were combined and dried, and the solvent was evaporated under reduced pressure to give compound **5** as yellow solid (Yield, 92%). ^1^H-NMR (300 MHz, DMSO-*d*_6_): δ 8.29 (1H, brd, *J* = 7.7 Hz), 7.74 (1H, brd, *J* = 7.7 Hz), 7.60 (1H, brt, *J* = 7.7 Hz), 7.38 (1H, brt, *J* = 7.7 Hz), 7.07 (2H, s, -NH_2_), 2.89 (2H, brt, *J* = 6.2 Hz), 2.55 (2H, brt, *J* = 6.2 Hz), 1.78–1.88 (4H, m); ESI-MS *m*/*z*: 199.1 [M + H]^+^ (C_13_H_14_N_2_).

#### 3.2.4. Procedure for the Preparation of 2-Chloro-*N*-(1,2,3,4-tetrahydroacridin-9-yl)acetamide (**6**)

A solution of chloroacetylchloride (0.5 mL) in CH_2_Cl_2_ (10 mL) was added dropwise to an ice-cold solution of triethylamine (2.5 mmol, 1.8 mL) and **5** (1.0 g, 5.0 mmol) in CH_2_Cl_2_ (30 mL). After the addition was completed, the solution was stirred for 2 h at room temperature. When the reaction was completed, it was diluted with CH_2_Cl_2_, washed with water, followed by brine solution. The organic layer was dried over anhydrous Na_2_SO_4_ and concentrated under reduced pressure. The obtained residue was purified by silica gel chromatography using CH_2_Cl_2_/MeOH (100:1) as eluent to give compound **6** as yellow solid (Yield, 65%). ^1^H-NMR (300 MHz, CDCl_3_): δ 8.42 (1H, s, -NH), 8.03 (1H, brd, *J* = 7.8 Hz), 7.78 (1H, brd, *J* = 7.8 Hz), 7.66 (1H, brt, *J* = 7.8 Hz), 7.51 (1H, brt, *J* = 7.8 Hz), 4.38 (2H, s), 3.17 (2H, brt, *J* = 6.4 Hz), 2.84 (2H, brt, *J* = 6.5 Hz), 1.99 (2H, m), 1.89 (2H, m); ESI-MS *m*/*z*: 279.2 [M + H]^+^ (C_15_H_15_ClN_2_O).

#### 3.2.5. Procedure for the Preparation of 2-(Piperazin-1-yl)-*N*-(1,2,3,4-tetrahydro -acridin-9-yl)acetamide (**7**)

A mixture of **6** (0.2 g, 0.7 mmol), anhydrous piperazine (0.9 g, 10.5 mmol) and KI (0.06 g, 0.4 mmol) in ethanol (30 mL) was refluxed for 1 h. After cooling, the solvent was removed under vacuum. The obtained residue was dissolved in CH_2_Cl_2_ and then washed with water. The organic phase was dried over anhydrous Na_2_SO_4_ and concentrated under reduced pressure. The crude product was purified by silica gel chromatography with CH_2_Cl_2_/MeOH (20:1) as eluent to afford compound **7** as yellow solid (Yield, 72%). ^1^H-NMR (300 MHz, CDCl_3_): δ 9.11 (1H, s, -NH), 8.01 (1H, brd, *J* = 8.0 Hz), 7.71 (1H, m), 7.65 (1H, m), 7.49 (1H, m), 3.46 (2H, brs), 3.16 (2H, brt, *J* = 6.3 Hz), 2.75–2.88 (6H, overlapped), 2.18 (4H, brs), 2.00 (2H, m), 1.90 (2H, m); ESI-MS *m*/*z*: 325.3 [M + H]^+^ (C_19_H_24_N_4_O).

#### 3.2.6. Procedure for the Preparation of TFAs **8a**–**e**

*Methyl (E)-3-(3-methoxy-4-(2-(4-(2-oxo-2-((1,2,3,4-tetrahydroacridin-9-yl)amino)ethyl)piperazin-1-yl)ethoxy)phenyl)acrylate* (**8a**): Compound **7** 100 mg (0.31 mmol) was dissolved in 50 mL dry acetonitrile, 120 mg (0.32 mmol) compound **3a** and 60 mg (1.4 mmol) anhydrous K_2_CO_3_ was added. The solution was refluxed for 12 h, and then filtered. The filtrate was concentrated under reduced pressure. The obtained residue was purified by silica gel chromatography with CH_2_Cl_2_/MeOH (30:1) as eluent to give compounds **8a** as white solid 0.114 g (Yield, 60%). ^1^H-NMR (600 MHz, CDCl_3_): δ 9.32 (1H, s, -NH), 8.03 (brd, 1H, *J* = 8.2 Hz), 7.72 (1H, brd, *J* = 8.2 Hz), 7.63 (1H, brt, *J* = 8.2 Hz), 7.60 (1H, d, *J* = 16.0 Hz), 7.47 (1H, brt, *J* = 8.2 Hz), 7.07 (1H, dd, *J* = 1.8, 8.2 Hz), 7.03 (1H, d, *J* = 1.8 Hz), 6.88 (1H, d, *J* = 8.2 Hz), 6.30 (1H, d, *J* = 16.0 Hz), 4.26 (2H, t, *J* = 6.2 Hz), 3.86 (3H, s), 3.78 (3H, s), 3.36 (2H, s), 3.14 (2H, t, *J* = 6.2 Hz), 3.01 (2H, t, *J* = 6.0 Hz), 2.85–2.97 (8H, brs), 2.80 (2H, t, *J* = 6.5 Hz), 1.95 (2H, m), 1.86 (2H, m); ^13^C-NMR (150 MHz, CDCl_3_): δ 168.3, 167.5, 159.5, 149.7, 149.5, 145.5, 144.5, 129.4, 128.1, 127.7, 127.2, 126.2, 124.5, 123.6, 122.3, 122.1, 115.9, 113.1, 110.1, 66.1, 61.3, 56.5, 55.8, 53.3, 52.9, 51.6, 33.2, 25.6, 22.3, 22.2; ESI-MS *m*/*z*: 559.3 [M + H]^+^ (C_32_H_38_N_4_O_5_).

The syntheses of compounds **8b**–**8e** were carried out by the same procedure as described for the preparation of **8a**.

*Methyl (E)-3-(3-methoxy-4-(3-(4-(2-oxo-2-((1,2,3,4-tetrahydroacridin-9-yl)amino)ethyl)piperazin-1-yl)propoxy)phenyl)acrylate* (**8b**): white solid 0.108 g (Yield, 59%). ^1^H-NMR (600 MHz, CDCl_3_): δ 9.61 (1H, s, -NH), 8.05 (brd, 1H, *J* = 8.3 Hz), 7.81 (1H, brd, *J* = 8.3 Hz), 7.65 (1H, brt, *J* = 8.3 Hz), 7.62 (1H, d, *J* = 15.7 Hz), 7.53 (1H, brt, *J* = 8.3 Hz), 7.09 (1H, dd, *J* = 2.1, 8.4 Hz), 7.04 (1H, d, *J* = 2.1 Hz), 6.88 (1H, d, *J* = 8.4 Hz), 6.31 (1H, d, *J* = 15.7 Hz), 4.15 (2H, t, *J* = 6.0 Hz), 3.88 (3H, s), 3.80 (3H, s), 3.44 (2H, s), 2.90-3.24 (12H, m), 2.83 (2H, t, *J* = 6.3 Hz), 2.30 (2H, m), 1.81–2.13 (4H, m); ^13^C-NMR (150 MHz, CDCl_3_): *δ* 168.2, 167.5, 159.0, 149.9, 149.4, 144.5, 143.5, 129.8, 128.1, 127.8, 127.7, 126.6, 124.6, 123.7, 122.6, 122.4, 115.7, 112.8, 110.1, 66.5, 61.0, 55.8, 54.9, 52.4, 51.6, 51.4, 32.5, 29.6, 25.5, 22.0, 21.9; ESI-MS *m*/*z*: 573.5 [M + H]^+^ (C_33_H_40_N_4_O_5_).

*Methyl (E)-3-(3-methoxy-4-(4-(4-(2-oxo-2-((1,2,3,4-tetrahydroacridin-9-yl)amino)ethyl)piperazin-1-yl)butoxy)phenyl)acrylate* (**8c**): white solid 0.092 g (Yield, 52%). ^1^H-NMR (600 MHz, CDCl_3_): δ 9.19 (1H, s, -NH), 8.02 (brd, 1H, *J* = 8.0 Hz), 7.72 (1H, brd, *J* = 8.0 Hz), 7.65 (1H, brt, *J* = 8.0 Hz), 7.63 (1H, d, *J* = 16.0 Hz), 7.49 (1H, brt, *J* = 8.0 Hz), 7.09 (1H, dd, *J* = 2.1, 8.2 Hz), 7.04 (1H, d, *J* = 2.1 Hz), 6.86 (1H, d, *J* = 8.2 Hz), 6.31 (1H, d, *J* = 16.0 Hz), 4.08 (2H, t, *J* = 6.3 Hz), 3.89 (3H, s), 3.80 (3H, s), 3.38 (2H, s), 3.16 (2H, t, *J* = 6.2 Hz), 2.94 (4H, brs), 2.82 (6H, overlapped), 2.67 (2H, m), 1.97 (2H, m), 1.78–1.94 (6H, m); ^13^C-NMR (150 MHz, CDCl_3_): δ 168.1, 167.5, 159.7, 150.3, 149.4, 144.6, 143.6, 129.0, 128.4, 127.4, 127.2, 126.0, 124.6, 123.6, 122.4, 121.9, 115.5, 112.4, 110.0, 68.7, 61.3, 57.5, 55.9, 52.6, 51.7, 51.3, 33.6, 29.6, 26.7, 25.7, 22.5, 22.3; ESI-MS *m*/*z*: 587.5 [M + H]^+^ (C_34_H_42_N_4_O_5_).

*Methyl (E)-3-(3-methoxy-4-((5-(4-(2-oxo-2-((1,2,3,4-tetrahydroacridin-9-yl)amino)ethyl)piperazin-1-yl)pentyl)oxy)phenyl)acrylate* (**8d**): white solid 0.101 g (Yield, 58%). ^1^H-NMR (600 MHz, CDCl_3_): δ 9.18 (1H, s, -NH), 8.02 (brd, 1H, *J* = 8.0 Hz), 7.71 (1H, brd, *J* = 8.0 Hz), 7.65 (1H, brt, *J* = 8.0 Hz), 7.63 (1H, d, *J* = 15.9 Hz), 7.49 (1H, brt, *J* = 8.0 Hz), 7.08 (1H, dd, *J* = 1.9, 8.2 Hz), 7.04 (1H, d, *J* = 1.9 Hz), 6.85 (1H, d, *J* = 8.2 Hz), 6.31 (1H, d, *J* = 15.9 Hz), 4.05 (2H, t, *J* = 6.4 Hz), 3.89 (3H, s), 3.80 (3H, s), 3.37 (2H, s), 3.15 (2H, t, *J* = 6.3 Hz), 2.94 (4H, brs), 2.81 (6H, overlapped), 2.58 (2H, m), 1.98 (2H, m), 1.83–1.94 (4H, m), 1.70 (2H, m), 1.54 (2H, m); ^13^C-NMR (150 MHz, CDCl_3_): δ 168.2, 167.6, 159.8, 150.5, 149.4, 146.5, 144.7, 128.9, 128.5, 127.3, 127.2, 125.9, 124.7, 123.6, 122.5, 121.8, 115.4, 112.3, 110.0, 68.5, 61.3, 57.8, 55.9, 52.7, 51.5, 51.3, 33.7, 29.6, 28.7, 25.7, 23.7, 22.5, 22.4; ESI-MS *m*/*z*: 601.6 [M + H]^+^ (C_35_H_44_N_4_O_5_).

*Methyl (E)-3-(3-methoxy-4-((6-(4-(2-oxo-2-((1,2,3,4-tetrahydroacridin-9-yl)amino)ethyl)piperazin-1-yl)hexyl)oxy)phenyl)acrylate* (**8e**): white solid 0.118 g (Yield, 61%). ^1^H-NMR (600 MHz, CDCl_3_): δ 9.21 (1H, s, -NH), 7.95 (brd, 1H, *J* = 8.2 Hz), 7.68 (1H, brd, *J* = 8.2 Hz), 7.59 (1H, brt, *J* = 8.2 Hz), 7.58 (1H, d, *J* = 15.5 Hz), 7.43 (1H, brt, *J* = 8.2 Hz), 7.03 (1H, dd, *J* = 1.9, 8.2 Hz), 7.00 (1H, d, *J* = 1.9 Hz), 6.81 (1H, d, *J* = 8.2 Hz), 6.26 (1H, d, *J* = 15.5 Hz), 4.00 (2H, t, *J* = 6.5 Hz), 3.84 (3H, s), 3.74 (3H, s), 3.27 (2H, s), 3.10 (2H, t, *J* = 6.5 Hz), 2.73–2.84 (6H, m), 2.55 (4H, brs), 2.34 (2H, brt, *J* = 6.7 Hz), 1.93 (2H, m), 1.78-1.86 (4H, m), 1.57 (2H, m), 1.46 (2H, m), 1.36 (2H, m); ^13^C-NMR (150 MHz, CDCl_3_): δ 168.6, 167.5, 159.7, 150.6, 149.4, 146.8, 144.7, 128.7, 128.6, 127.1, 126.9, 125.8, 124.7, 123.6, 122.4, 121.8, 115.2, 112.2, 110.0, 68.7, 61.7, 58.3, 55.9, 53.9, 53.2, 51.5, 33.9, 28.9, 27.1, 26.7, 25.9, 25.6, 22.6, 22.4; ESI-MS *m*/*z*: 615.5 [M + H]^+^ (C_36_H_46_N_4_O_5_).

### 3.3. AChE and BuChE Inhibitory Activity Assay

Five different concentrations (10^−4^–10^−9^ M) of each compound were tested for the inhibitory activity for AChE and BuChE. 10 μL 0.86 U/mL AChE or BuChE, 60 μL 0.1 M phosphate buffer (pH 8.0), and 10 μL test compounds were incubated together at 37 °C for 10 min. An amount of 20 μL 10 mmol/L acetylcholine iodide or butylthicoline iodide was added and the solution was incubated at 37 °C for 10 min again. Then 20 μL 5 mmol/L 5,5′-dithiobis-(2-nitrobenzoic acid) (DTNB) and 30 μL 1% sodium dodecyl sulfatesodium salt (SDS) were added. Optical density (OD) values were tested at 415 nm and the percent inhibitions were calculated. Each concentration was analyzed three times, and the IC_50_ value was calculated from the concentration-inhibition response curve on logarithmic scale.

### 3.4. Inhibition of Aβ (1–42) Self-Induced Aggregation Assay

An amount of 250 μg Aβ (1–42) was dissolved in 1 mL hexafluoroisopropanol (HFIP) and lyophilizated, then dissolved in 25 μL DMSO and 150 μL 50 mM phosphate buffer saline (PBS). For the self-induced assay, 10 μL of Aβ (1–42) was incubated with 10 μL test compounds, both at a final concentration of 20 μM, at 37 °C for 48 h. Then, 40 μL PBS and 100 μL 5 μM thioflavin T (ThT) in 50 mM glycine-NaOH buffer (pH 8.5) was added. Fluorescence was monitored at 450 nm and emission 482 nm using a microplate reader (BioTek Synergy H1, BioTek Instruments, Inc., Winooski, VT, USA). The percent inhibition of the self-induced aggregation due to the presence of the test compound was calculated by the following expression: 100 − (IF_i_/IF_0_ × 100) where IF_i_ and IF_0_ are the fluorescence intensities with and without test compound, respectively, minus the fluorescence intensities due to the respective blanks. 

### 3.5. Cu^2+^-Chelating Properties Assay

The complexing studies were determined using a UV spectrophotometer (Beckman Coulter Inc., Brea, CA, USA). Compound **8d** (40 µM) in methanol was mixed with 10, 20, 40 µM CuCl_2_, and then incubated at room temperature for 5 min. The absorption spectra were recorded from 220 nm to 400 nm after incubation.

### 3.6. Kinetic Analysis of AChE Inhibition

Kinetic characterization of AChE was performed as the reported method using six different substrate concentrations (50, 90, 150, 266, 452, and 904 μM). An amount of 60 µL PBS, 20 µL DTNB, 10 µL AChE, and 10 µL inhibitor were mixed together and pre-incubated for 10 min, followed by the addition of 20 μL different substrate and then incubated for 15 min. After that, 30 μL 1% SDS was added. Kinetic characterization of the hydrolysis of acetylthiocholine chloride catalyzed by AChE was done spectrometrically at 415 nm. A parallel control with no inhibitor in the mixture, allowed adjusting activities to be measured.

### 3.7. Cell Culture and Viability Assay

Neuro-2A cell were grown in dulbecco’s modified eagle medium (DMEM) plus 1% glutamine and 10% heat-inactivated fetal bovine serum. The assays were performed in 96-well microtiter plates. Cells were treated with or without lyophilizated Aβ (1–42) (10 μM), or different concentrations of **8d** (5, 10 μM) combination and incubated for 48 h. Then, 10 µL of MTT (5 mg/mL) was added to each well and incubated for four hours, and then the liquid in the wells was removed. DMSO (150 µL) was added to each well. The absorbance was recorded using a microplate reader at a wavelength of 570 nm. 

## 4. Conclusions

In summary, we have designed and synthesized five novel hybrid compounds (**8a**–**e**) including tacrine, ferulic acid moiety, and piperazine fragment. All compounds effectively inhibited cholinesterase (ChE), reduced self-induced β-amyloid (Aβ) aggregation and chelated Cu^2+^ in vitro. At the same time, it exhibited the protective effect of TFAs on the Aβ-induced neurotoxicity. The above results suggested that these compounds are potential candidates for treating AD.

## Figures and Tables

**Table 1:** Inhibition of ChEs activity and self-induced Aβ (1–42) aggregation.

	Compound	IC_50_ (nM) AChE ^a^	IC_50_ (nM) BuChE ^b^	Selectivity Index ^c^	Aβ (1–42) Aggregation Inhibition (%) ^d^
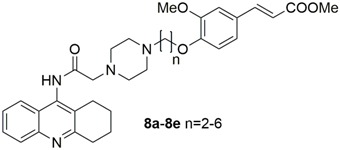	**8a**	306.5 ± 12.4	202.9 ± 35.7	0.660	26.1 ± 4.5
	**8b**	90.3 ± 7.3	119.7 ± 12.5	1.326	19.5 ± 2.7
	**8c**	52.7 ± 8.2	215.4 ± 33.7	4.087	18.5 ± 4.0
	**8d**	61.7 ± 5.2	106.9 ± 13.1	1.733	37.2 ± 0.9
	**8e**	184.1 ± 15.2	195.7 ± 3.5	1.059	35.1 ± 3.2
	tacrine	151.1 ± 10.3	27.6 ± 7.3	0.182	n.t. ^e^
	curcumin	n.t.	n.t.	n.t.	37.3 ± 1.2
	ferulic acid	n.t.	n.t.	n.t.	36.5 ± 3.6

^a^ Inhibitor concentration (mean ± SD of three independent experiments) required for 50% inactivation of AChE. ^b^ Inhibitor concentration (mean ± SD of three independent experiments) required for 50% inactivation of BuChE. ^c^ Selectivity index = IC_50_ (BuChE)/IC_50_ (AChE). ^d^ Inhibition of self-induced Aβ (1–42) aggregation, the thioflavin-T fluorescence method was used, the mean ± SD of at least three independent experiments and the measurements were carried out in the presence of 20 μM compounds. ^e^ n.t. = not tested.
